# Supplementary material for: Natural variations in the biofilm-associated protein BslA from the genus Bacillus
Source: Sci Rep. 2017 Jul 27;7:6730. doi: 10.1038/s41598-017-06786-9 (PMC5532214; doi:10.1038/s41598-017-06786-9)
Supplement: Supplementary file 1 — Supplementary Info [file 41598_2017_6786_MOESM1_ESM.pdf]

Natural variations in the biofilm-associated protein BslA from the genus *Bacillus*

Ryan J. Morris[a], Marieke Schor [a], Rachel M.C. Gillespie[b], Ana Sofia Ferreira[b], Lucia Baldauf[a], Chris Earl[b], Adam Ostrowski[b], Laura Hobley[c], Keith M. Bromley[a], Tetyana Sukhodub[b], Sofia Arnaouteli[b], Nicola R. Stanley-Wall[b], and Cait E. MacPhee[a]

[a]James Clerk Maxwell Building, School of Physics, University of Edinburgh, Edinburgh EH9 3JZ, United Kingdom, [b]Division of Molecular Microbiology, School of Life Sciences, University of Dundee, Dundee DD1 5EH, United Kingdom, [c]School of Medicine, Dentistry, and Biomedical Sciences, Queen's University Belfast

Table S1: Full list of strains used in this study.

Strain	Relevant genotype/Description[a]	Source/Construction[b,c]
MC1061	E. coli F'lacIQ lacZM15 Tn10 (tet)	E. coli Genetic Stock Centre
BL21 (DE3)	F-ompT hsdSB(rB–, mB–) gal dcm (DE3)	[3]
NCIB3610	prototroph	BGSC
168	trpC2	BGSC
NRS2404	168 yweA::kan	NSW806/NSW809 → 168
NRS2405	3610 yweA::kan	SPP1 NRS2404 → 3610
NRS2406	3610 bslA::cat yweA::kan	SPP1 NRS2404 → NRS2097
NRS2409	168 amyE::Phy-spank-yweA-lacI (spc)	pNW608 → 168
NRS2412	NCIB3610 bslA::cml amyE::Phy-spank-yweA-lacI(spc)	SPP1 NRS2409 → NRS2097
NRS2097	NCIB3610 bslA::cml	[4]
NRS2299	NCIB3610 bslA::cml amyE::Phy-spank-bslA-lacI	[4]
NRS2410	168 amyE::Phy-spank-bslA$_{B_lic}$-lacI (spc)*	pNW609 → 168
NRS2412	NCIB3610 bslA::cml amyE::Phy-spank-bslA-lacI	SPP1 NRS2409→NRS2097
NRS2414	NCIB3610 bslA::cml amyE::Phy-spank-bslA$_{B_lic}$-lacI	SPP1 NRS2410→NRS2097
NRS2456	168 amyE::Phy-spank-bslA$_{B_amy}$-lacI (spc)**	pNW615→168
NRS2458	NCIB3610 bslA::cml amyE::Phy-spank-bslA$_{B_amy}$-lacI	SPP1 NRS2456→NRS2097
NRS2462	168 amyE::Phy-spank-bslA$_{B_pum}$-lacI (spc)***	pNW620→168
NRS2464	NCIB3610 bslA::cml amyE::Phy-spank-bslA$_{B_pum}$-lacI	SPP1 NRS2462→NRS2097
NRS4264	168 lacA::PsspB-yfp	[5]
NRS5188	NCIB3610 lacA::PsspB-yfp (mls)	SPP1 NRS4264→3610
NRS5186	NCIB3610 bslA::cml lacA::PsspB-yfp (mls)	NRS4264→NRS2097
NRS5187	NCIB3610 bslA::cml amyE::Phy-spank-bslA-lacI (spc) lacA::PsspB-yfp (mls)	SPP1 NRS4264→NRS2299
NRS5199	NCIB3610 bslA::cml amyE::Phy-spank-bslA$_{B_lic}$-lacI (spc) lacA::PsspB-yfp (mls)	SPP1 NRS4264→2414
NRS5206	NCIB3610 bslA::cml amyE::Phy-spank-bslA$_{B_amy}$-lacI (spc) lacA::PsspB-yfp (mls)	SPP1 NRS4264→2458
NRS5205	NCIB3610 bslA::cml amyE::Phy-spank-bslA$_{B_pum}$-lacI (spc) lacA::PsspB-yfp (mls)	SPP1 NRS4264→NRS2464
NRS5659	168 amyE::Phy-spank-bslA$_{B_licG152S}$-lacI(spc)*	pNW1636 → 168
NRS5662	NCIB3610 bslA::cml amyE::Phy-spank-bslA$_{B_licG152S}$-lacI(spc)*	SPP1 NRS5659 → NRS2097
NRS5660	168 amyE::Phy-spank-bslA$_{B_licA76SG152S}$-lacI(spc)*	pNW1637 → 168
NRS5663	NCIB3610 bslA::cml amyE::Phy-spank-bslA$_{B_licA76SG152S}$-lacI(spc)*	SPP1 NRS5660 → NRS2097
NRS5661	168 amyE::Phy-spank-bslA$_{B_licA76S}$-lacI(spc)*	pNW1478 → 168
NRS5664	NCIB3610 bslA::cml amyE::Phy-spank-bslA$_{B_licA76S}$-lacI(spc)*	SPP1 NRS5661 → NRS2097

a. Drug resistance cassettes are indicated as follows: *cml*, chloramphenicol resistance; *mls* lincomycin and erythromycin resistance and *spc*, spectinomycin resistance.
b BSGC represents the *Bacillus* genetic stock centre.
c. The direction of strain construction is indicated with DNA or phage (SPP1) (→) recipient strain.
*Allele of bslA amplified from B. licheniformis DSM13; **Allele of bslA amplified from B. amyloliquefaciens FZB42; ***Allele of bslA amplified from B. pumilus SAFR-032.

Table S2: Oligonucleotide primers used in this study

Primer	Sequence 5' to 3'[a]	Use
NSW152	CAGCGAACCATTTGAGGTGATAGG	*kan* cassette amplification
NSW153	CGATACAAATTCCTCGTAGGCGCTCGG	*kan* cassette amplification
NSW806	CCAAAAGGGGTGGCTCACGGATATAGAGTGC	*yweA* deletion
NSW807	CCTATCACCTCAAATGGTTCGCTGTTTTAGCATGACATTTCC	*yweA* deletion
NSW808	CGAGCGCCTACGAGGAATTTGTATCGATCCCCGTTAATCG	*yweA* deletion
NSW809	AATCCCGCATTTGAGCGTCATCC	*yweA* deletion
NSW810	GCTCAAGCTTCAATTAGGGGGAAATGTCATGCTAAAA	*yweA* cloning
NSW811	GCATGCATGCCAAATCTATCGATTAACGGG	*yweA* cloning
NSW812	GCAAAAGCTTTTGGGGGAATTGCTGTGTTG	*bslA B_ lic* cloning*
NSW813	CGTAGCATGCTTGCCTATTTACTTGCAG	*bslA B_ lic* cloning
NSW819	GCTTAAGCTTGGGGGAAGGAAGAAATGAAAAAAAC	*bslA B_ pum* cloning**
NSW820	GCTTAAGCTTGGGGGAAGGAAGAAATGAAAAAAAC	*bslA B_ pum* cloning
NSW829	GCATGTCGACTTTTAGGGGGATTTATGAAAATG	*bslA B_ amy* cloning***
NSW830	CGTAGCATGCGGTCTTTTTTTCGCAATTATTTGC	*bslA B_ amy* cloning
NSW1853	GTACCATATGCAGTCTGCATCAATCGAG	$yweA_{31-155}$ cloning
NSW1854	GATCCTCGAGTTATTAACGGGGATCAATCAC	$yweA_{31-155}$ cloning
NSW2011	CAGGGGCCCCTGGGATCCGAAAATTTATATTTTCAAATGTCCACGAA AGCGACTGC	*bslA B_* amy_{42-181} cloning
NSW2012	GCATCTCGAGTTATTATTTGCAGTTGCAAGGCTGTGTCG	*bslA B_* amy_{42-181} cloning
NSW2013	CAGGGGCCCCTGGGATCCGAAAATTTATATTTTCA ATACCGCCCGGCTGCG	*bslA B_* lic_{40-179} cloning
NSW2014	GCATCTCGAGTTATTACTTGCAGTTGCACGGC	*bslA B_* lic_{40-179} cloning
NSW2015	CAGGGGCCCCTGGGATCCGAAAATTTATATTTTCA ATCAACAAACGCCAGACCAGC	*bslA B_* pum_{37-177} cloning
NSW2016	GCATCTCGAGTTATTAGCAACCACACGGATCTGG	*bslA B_* pum_{37-177} cloning
NSW2036	GTCCTAACTCAATCTTG**agc**CTGGGAACGGTTGAA	*B_ lic A76S*
NSW2037	TTCAACCGTTCCCAG**gct**CAAGATTGAGTTAGGAC	*B_ lic A76S*
NSW2038	GAGAACAGAGTACTG**agc**CTCGGATCGACTTTC	*B_ lic G152S*
NSW2039	GAAAGTCGATCCGAG**gct**CAGTACTCTGTTCTC	*B_ lic G152S*
NSW2191	5' GATATGTTAACTCAATAT 3'	*Q5 mutagenesis B_ lic A76S*

a Restriction sites are underlined and sites for mutations are highlighted in lower case bold.
*Allele of *bslA* amplified from *B. licheniformis* DSM13; **Allele of *bslA* amplified from *B. pumilus* SAFR-032; ***Allele of *bslA* amplified from *B. amyloliquefaciens* FZB42.

Table S3: Plasmids used in this study

Plasmid	Description	Source
pDR111	*B. subtilis* integration vector for IPTG-induced expression	[1]
pGEX-6P-1	Vector for overexpression of GST-fused proteins	GE Healthcare
pNW608	pDR111-*yweA*	This work
pNW609	pDR111-$bslA_{B\_lic}$*	This work
pNW615	pDR111-$bslA_{B\_amy}$**	This work
pNW620	pDR111-$bslA_{B\_pum}$***	This work
pNW1420	pET15bTEV-$ywea_{31-155}$	This work
pNW1128	pGEX-6P-1-TEV-$bslA_{42-181}$	[2]
pNW1422	pGEX-6P-1-TEV-$bslA_{B\_amy\_42-181}$	This work
pNW1423	pGEX-6P-1-TEV-$bslA_{B\_lic\_40-179}$	This work
pNW1424	pGEX-6P-1-TEV-$bslA_{B\_pum\_37-177}$	This work
pNW1463	pGEX-6P-1-TEV-$bslA_{B\_lic\_40-179\ A76S}$	This work
pNW1464	pGEX-6P-1-TEV-$bslA_{B\_lic\_40-179\ G152S}$	This work
pNW1465	pGEX-6P-1-TEV-$bslA_{B\_lic\_40-179\ A76S\ G152S}$	This work
pNW1478	pDR111-$bslA_{B\_lic\_A76S}$	This work
pNW1636	pDR111-$bslA_{B\_lic\_G152S}$	This work
pNW1637	pDR111-$bslA_{B\_lic\_A76SG152S}$	This work

*Allele of *bslA* amplified from *B. licheniformis*; **Allele of *bslA* amplified from *B. amyloliquefaciens* FZB42; ***Allele of *bslA* amplified from *B. pumilus* SAFR-032.

Bacterial Species	BslA	YweA
Bacillus subtilis	AIY94428.1	NP_391660.1
Bacillus mojavensis	WP_024122618.1	WP_024123256.1
Bacillus licheniformis	AAU25437.1	AAU25542.1
Bacillus sonorensis	EME73570.1	EME73667.1
Bacillus tequilensis	WP_024713035.1	WP_024713112.1
Bacillus vallismortis	WP_010329773.1	WP_061572196.1
Bacillus atrophaeus	ADP33594.1	ADP34282.1
Bacillus amyloliquefaciens	CBI44000.1	CBI44741.1
Bacillus siamensis	OAZ60821.1	N/D
Bacillus pumilus	ABV62292.1	N/D

Table S4 Accession numbers for BslA and YweA protein sequences from a range of *Bacillus* species used in this study for bioinformatics analysis. N/D indicated not detected.

```
B_PUM_BSLA  --MKK-TWTMIMMGMLTLVMALSVPIAASAEGAT----QEGKASTNARPAELYAKITGTSKQEWS 058
B_LIC_BSLA  --MKRMYRSKLSILAVSLVMMASIFLPSFQASAQTT--KTESVYRPAASASLYSVITGASKQEWS 061
B_SON_BSLA  --MKNMFRSTLTMMMAGFVIMASMFLPSFHADAKTV--KTESVNRPAAKASLHSVITGASKQEWS 061
B_TEQ_BSLA  --MKRKLLSSLAISALSLGLLVSAPTASFAAESTATKAHTESTMKTQATASLFATITGASKTEWS 063
B_SUB_BSLA  --MKRKLLSSLAISALSLGLLVSAPTASFAAESTSTKAHTESTMRTQSTASLFATITGASKTEWS 063
B_VALL_BSLA --MKRKLLSSLAISALSLGLLVSAPTASFAAESKSTSAHTESTMKTQSTASLFATITGTSKTEWS 063
B_MOJ_BSLA  --MKRKLISSLAISVLSLGLLVSAPSASFAAESKSSSAHTESTMRAQSTASLFATITGASKTEWS 063
B_ATRO_BSLA --MKHKLFASLITSLLGLVLLMAVPATSFAAESKSA--ANEPTMHTTATASLFAKITGTSKQEWS 061
B_AMY_BSLA  MKMKHKFFSTVMASLFGLVLLLSLPTASFAAESSST--VHEPEMSTKATATLFAKYTGASQQEWS 063
B_SIAM_BSLA --MKQKFFSTVMASLFGLALLLSLPTASFAAESGST--VHEPEMSTKATATLFAKYTGASQQEWS 061
B_LIC_YWEA  -MLKRKFIGKIGVGLLTSAALFSFILPTEEASATFYR----------NAP-TLHVETVDSNKEWT 053
B_SON_YWEA  -MLKRNIISKISIGLLTSAALFSFILPSQEAHATFFR----------SEP-TLHVETVDSNKEWT 053
B_AMY_YWEA  MMLKRKSF---SAFFISAAVFLAVFLPSFHANAQ--------------SA-VIEAKTINSTKEYA 047
B_ATRO_YWEA -MLKRTSL---FSVFISSAVLLSILLPSVHANAK--------------SA-SIEAKTINSTKEYA 046
B_VALL_YWEA -MLKRTSL---FSVFISSAVLLSILLPSVHANAQ--------------SA-SIEAKTINSTKEYA 046
B_TEQ_YWEA  -MLKRTSF--VSSLFISSAVLLSILLPSGHAHAK--------------SA-SIGAKTINSTTEWT 047
B_SUB_YWEA  -MLKRTSF--VSSLFISSAVLLSILLPSGQAHAQ--------------SA-SIEAKTVNSTKEWT 047
B_MOJ_YWEA  -MLKRNSF--VSSFFISSAVLLSILLPSGQAHAQ--------------SA-SIEAKTVNSTKEWT 047
```

```
                         *
B_PUM_BSLA  FSDIELTYRPNSVLSLGAIEFTLPAGFQATTKDIFNGKALKDSYILNSGKTVRIPARLDLLGISQ 123
B_LIC_BSLA  FSDIELTYRPNSILALGTVEFTLPSGFSATTKDTVNGRALTTGQILNNGKTVRLPLTIDLLGIAE 126
B_SON_BSLA  FSDIELTYRPNSILALGTVEFTLPSGFSATTKDTVNGRALTTGQILNNGKTVRLPLTIDLLGIAE 126
B_TEQ_BSLA  FSDIELTYRPNTLLSLGVMEFTLPSGFTANTKDTMNGHALRTTQILNNGKTVRIPLALDLLGAGE 128
B_SUB_BSLA  FSDIELTYRPNTLLSLGVMEFTLPSGFTANTKDTLNGNALRTTQILNNGKTVRVPLALDLLGAGE 128
B_VALL_BSLA FSDIELTYRPNTLLSLGVMEFTLPSGFTANTKDTMNGRALRETQILNNGKTVRIPLALDLLGAGE 128
B_MOJ_BSLA  FSDIELTYRPNTLLSLGVMEFTLPSGFTANTKDTMNGNALRETQILNNGKTVRVPLALDLLGAGE 128
B_ATRO_BSLA FSDIELTYRPNTILSLGVMEFTLPSGFTATTKDFVNGHALRERQILNNGKTVRLPLNLDLLGAAE 126
B_AMY_BSLA  FSDIELTYRPNTILSLGVMEFTLPSGFTATTKDTVNGHALRERQILNNGKTVRLPLNIDLIGAAE 128
B_SIAM_BSLA FSDIELTYRPNTILSLGVMEFTLPSGFAATTKDTINGHALRERQILNNGKTVRLPLNIDLLGAAE 126
B_LIC_YWEA  TSDIEVTYKPNFFVGSSYVEFNFPYRFHANTRDSLNGRTLNYTQILNDGQTVRVPVY--AFSSSE 116
B_SON_YWEA  TSDIEVTYKPNFFVGASYVEFNFPYRFHANTRDSLNGRTLNYTQILNDGQTVRVPVY--AFSSSQ 116
B_AMY_YWEA  TSDIEVTYKPNALLAVGAIEFQFPDGFNATVRDSVNGRTLKETQILNYGKTVRLPLTLDLLGATE 112
B_ATRO_YWEA TSDIEVTYRPKAVLSLGTIEFNFPDGFHANTRDSINGKTLKETQILNDGKKVRLPFTLDLLGAAE 111
B_VALL_YWEA TSDIEVTYRPKAVLSLGTIEFNFPDGFHANTRDSINGKTLKETQILNDGKKVRLPFTLDLLGAAE 111
B_TEQ_YWEA  TSDIEVTYKPKAVFSLGAVEFRFPDGFHATTRDSVNGRTLKETQILNDGKTVRLPLTLDLLGRSE 112
B_SUB_YWEA  ISDIEVTYKPNAVLSLGAVEFQFPDGFHATTRDSVNGRTLKETQILNDGKTVRLPLTLDLLGASE 112
B_MOJ_YWEA  TSDIEVTYKPNAVLSLGAVEFQFPDGFHATTRDSVNGRTLKETQILNDGKTVRLPLTLDLLGASE 112
```

```
                                    *
B_PUM_BSLA  FKLQLSHKVLPAAGTYTFRAEN-RALSIGSKFYAEDTLDIQTRPVVVTPPDPCGC- 177
B_LIC_BSLA  FKLVLANKTLPAAGKYTFRAEN-RVLGLGSTFYAESSIEVQKRAT--PPTQPCNCK 179
B_SON_BSLA  FKLVLANKTLPAAGKYTFRAEN-RVLGLGSTFYAESSIEVQKRST--PPTQPCNCN 179
B_TEQ_BSLA  FKLRLNNKTLPAAGTYTFRAEN-RSLSVGDRFYAEASIDVAKRST--PPTQPCDSK 181
B_SUB_BSLA  FKLKLNNKTLPAAGTYTFRAEN-KSLSIGNKFYAEASIDVAKRST--PPTQPCGCN 181
B_VALL_BSLA FKLKLNNKTLPAAGTYTFRAEN-KSLSIGNKFYAEASIDVAKRST--PPTQPCGCN 181
B_MOJ_BSLA  FKLKLNNKTLPAAGTYTFRAEN-KSLSIGNKFYAEASIEVAKRST--PPTQPCGCK 181
B_ATRO_BSLA FKLKLNNKTLPAAGTYTFRAEN-KSLSIGSKFYAEDSIVVEKRST--PPTKPCNCN 179
B_AMY_BSLA  FKLSLNNKTLPAAGTYKFRAEN-KSLSIGSKFYAEDTIVVQKRST--PPTQPCNCK 181
B_SIAM_BSLA FKLSLNNKTLPAAGTYKFRAEN-KSLSIGSKFYAEDTIVVQKRST--PPTQPCNCK 179
B_LIC_YWEA  FKLVMVRKTLPNAGTHRITAEL-QKFGRN-YNHAEATVDILPR------------- 157
B_SON_YWEA  FKLVMVRKTLPNAGTHRVTAEL-QKFGRH-YHHAEATVEIAPR------------- 157
B_AMY_YWEA  YNLVLVRKNLPRAGTYTIKGDYVNGLGAG-SLYAETKLVIDPR------------- 154
B_ATRO_YWEA FELVMVRKNLPRAGTYTIKGDMVNGLGIG-SFYAETQLVIDPR------------- 153
B_VALL_YWEA FELVMVRKNLPRAGTYTIKGDMVNGLGIG-SFYAETQLVIDPR------------- 153
B_TEQ_YWEA  FDLVMVRKTLPRAGTYTIKGNVVNGFGIG-SFYAETQLVIDPR------------- 154
B_SUB_YWEA  FDLVMVRKTLPRAGTYTIKGDVVNGLGIG-SFYAETQLVIDPR------------- 154
B_MOJ_YWEA  FDLVMVRKTLPRAGTYTIKGDVVNGLGIG-SFYAETQLVIDPR------------- 154
```

Figure S1: Amino acid alignment of BslA and YweA variants found in other Bacillus species. Abbreviations used are as follows: B_sub, *Bacillus subtilis*; B_pum, *Bacillus pumilis*; B_amy, *Bacillus amyloliquefaciens*; B_lic, *Bacillus licheniformis*; B_son, *Bacillus sonorensis*; B_teq, *Bacillus tequilensis*; B_vall, *Bacillus vallismortis*; B_atro, *Bacillus atrophaeus*; B_siam, *Bacillus siamensis*; B_moj, *Bacillus mojavensis.* The variants that are bolded and underlined were studied in this work. Underlined and bolded amino acids signify the signal sequence, black represents 100% sequence identity. Blue amino acids represent the hydrophobic cap regions, where dark blue are conserved amino acids. The purple highlighted amino acids are glycine which are conserved across nearly all species and follow the amino acids comprising the caps. Aspartic acid is shown in red and serine residues within caps 1 and 2 are shown in green. Note that YweA is differentiated from Bs_BslA and the BslA orthologues by the fact it lacks both the N-terminal region following the signalling sequence and the C-terminal domain. The * symbols indicate the cap regions containing serine residues.

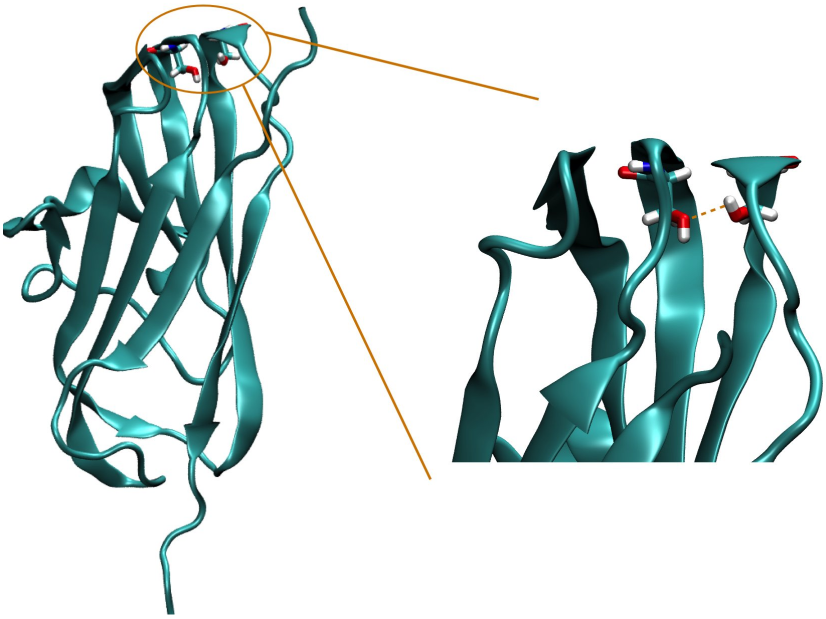

Figure S2: Orientation of serine residues. A depiction of Chain C of BslA derived from the crystal structure PDB: 4BHU. The conformation of Chain C corresponds to the the interfacial conformation of BslA. The zoomed in view highlights the orientation of serine residues within the cap and the dashed line between residues indicates they are within a distance where predicted hydrogen bonding can take place. Images generated using Visual Molecular Dynamics [6]

References

[1] Britton RA, *et al.* (2002) Genome-Wide Analysis of the Stationary-Phase Sigma Factor (Sigma-H) Regulon of *Bacillus subtilis*. *J Bacteriol* 184(17):4881-890.

[2] Hobley L, *et al.* (2013) BslA is a self-assembling bacterial hydrophobin that coats the Bacillus subtilis biofilm. *Proc Natl Acad Sci U S A* 110:13600-13605.

[3] Studier FW, Moffatt BA (1986) Use of bacteriophage T7 RNA polymerase to direct selective high-level expression of cloned genes. *J Mol Biol* 189(1):113-30.

[4] Verhamme DT, Murray EJ, Stanley-Wall NR (2009) DegU and Spo0A jointly control transcription of two loci required for complex colony development by *Bacillus subtilis*. *J Bacteriol* 191(1):100-08.

[5] Marlow, V. L., Porter, M., Hobley, L., Kiley, T. B., Swedlow, J. R., Davidson, F. A., Stanley-Wall, N. R. (2014). Phosphorylated DegU manipulates cell fate differentiation in the *Bacillus subtilis* biofilm. *J. Bacteriol*, 196(1): 16-27.

[6] Humphrey W, Dalke A, Schulten K (1996). VMD: Visual molecular dynamics. *J Mol Graph* 14(1):33-38, 27-28 .
